# Creatine monohydrate versus creatine hydrochloride on strength and body composition in elite team-sport athletes: A placebo-controlled randomized clinical trial comparing low dosages

**DOI:** 10.1080/15502783.2025.2533658

**Published:** 2025-07-16

**Authors:** Daniel Londoño-Velásquez, Yuliana Zuluaga-Narváez, Luis Rojas-Posada, Maximiliano Kammerer-López, Óscar M. Cardona-Arenas, Olga L. Quiroz-Bastidas, Mario A. Quintero-Velásquez, Andrés Rojas-Jaramillo, Richard B. Kreider, Diego A. Bonilla

**Affiliations:** a Grupo de Investigación NUTRAL, Facultad Ciencias de la Nutrición y los Alimentos, Universidad CES, Medellín, Colombia; b Grupo de Investigación CINDA, Instituto Departamental de Deportes de Antioquia (INDEPORTES), Medellín, Colombia; c Grupo de Investigación GRICAFDE, Instituto Universitario de Educación Física y Deporte (IUEFD), Universidad de Antioquia, Medellín, Colombia; d Research Division, Dynamical Business & Science Society — DBSS International SAS, Bogotá, Colombia; e Exercise & Sport Nutrition Laboratory, Human Clinical Research Facility, Texas A&M University, College Station, USA; f Hologenomiks Research Group, Department of Genetics, Physical Anthropology and Animal Physiology, University of the Basque Country (UPV/EHU), Leioa, Spain

**Keywords:** Creatine, randomized controlled trial, muscle strength, body composition, athletes, dietary supplements

## Abstract

**Background:**

Elite athletes often use creatine supplements to boost strength and improve body composition. While creatine monohydrate (CrM) is well-established, creatine hydrochloride (CrHCl) has gained attention for its claims of superior effects, especially at lower effective doses. This three-arm triple-blind placebo-controlled randomized clinical trial aimed to compare the effects of low-dose CrM and CrHCl supplementation on strength and body composition in elite team-sport athletes.

**Methods:**

A total of 31 male and female team-sport athletes (handball and softball players), aged 18–28 years, were randomly assigned to consume 5 g/day of creatine monohydrate (CrM) (*n* = 11), creatine hydrochloride (Cr-HCl) (*n* = 10), or placebo (maltodextrin) (*n* = 10). All groups followed an 8-week supplementation protocol with standardized body composition and performance tests assessed pre- and post-intervention. The isokinetic strengths and peak torque of the external and internal rotation muscle strengths of the bilateral shoulders were measured using a Humac Norm isokinetic dynamometer (Computer Sports Medicine Inc., Stoughton, MA, USA). The countermovement jump (CMJ) test was performed on a jump mat (Smart Jump; Fusion Sport, Coopers Plains, Australia) while the drop jump (DJ) was measured using a uniaxial force platform (ForceDecks, VALD). The Reactive Strength Index (RSI) was calculated as the ratio of flight time to contact time during the landing. Whole- and regional body composition were estimated using dual-energy x-ray absorptiometry (DXA). Adjusted fat-free mass (FFM) and FFM index (FFM/stature^2^) were derived. ClinicalTrials.gov ID #NCT05697900.

**Results:**

Comparison of neuromuscular and body composition variables between groups revealed no statistically significant differences in any measure. Within-group analysis showed significant improvements (P < 0.05) in jump performance for both CrM and Cr-HCl, with similar effect sizes (*d_unb_* < 0.3). Both creatine groups (Cr-HCl and CrM) also exhibited significant improvements in FFM (P < 0.05), although only the CrM group showed an increased FFM index.

**Conclusions:**

CrM and Cr-HCl supplementation produced similar effects on neuromuscular and strength performance in handball and softball players. Low doses of both creatine forms appeared effective for enhancing body composition, although only the CrM group demonstrated significant improvements in FFM index. Based on our findings, claims of Cr-HCl superiority are unfounded and misleading, as this form of creatine does not outperform CrM even at low doses in elite team-sport athletes.

**Disclosures:**

RBK serves as Chair of the “Creatine for Health” scientific advisory board sponsored by Creapure® and Creavitalis®—Alzchem Group AG, while DAB serves as a member of this board. DAB also acts as Scientific and Managing Director of KreaFood, an R&D&I project, and leads the CREAS project by DBSS (available at: http://CREAS.pro/).

